# COVID-19 and Biomarkers: The Contribution of the Journal

**DOI:** 10.3390/jcm12051853

**Published:** 2023-02-26

**Authors:** Mario Plebani

**Affiliations:** 1 Clinical Biochemistry and Clinical Molecular Biology, University of Padova, 35122 Padova, Italy; mario.plebani@unipd.it; 2 Department of Pathology, University of Texas, Medical Branch, Galveston, TX 77550, USA

Three years after coronavirus disease 2019 (COVID-19) was declared a pandemic by the World Health Organization (WHO), several features of the pathogenesis and innate immune response to SARS-CoV-2 (severe acute respiratory syndrome coronavirus disease 2) have now been clarified. Several studies have highlighted the crucial interplay among inflammation, thrombosis, cardiovascular diseases, multi-visceral manifestations, and SARS-CoV-2 infection, namely, linking the roles of cytokines, neutrophil extracellular traps (NETs), nucleosomes, histones, and the coagulation cascade. Although dramatic improvements have been achieved in better understanding the pathophysiology of the disease, the mechanisms of innate immune response and more appropriate therapeutic treatments, reliable and accurate biomarkers for the early risk stratification and clinical management of COVID-19 patients are still lacking. A series of papers recently published in the *Journal* have provided new insights into some interesting and potentially useful biomarkers in COVID-19. The first paper is by Simon T-P et al., who evaluated the prognostic value of bioactive adrenomedullin (bio-ADM) in critically ill patients with COVID-19. The rationale of the study, as underlined by the authors, is that “endothelial cell infection, inflammation and activation with the subsequent interruption of endothelial barrier function have been described in COVID-19 patients and adrenomedullin (ADM) has been shown to play a key role in regulating vascular hyper- permeability and endothelial stability/integrity in patients with severe infection” [1].

Adrenomedullin (ADM) is a free-circulating 52 amino-acid peptide belonging to the calcitonin gene-related peptide family that was first discovered in human pheochromocytoma tissue three decades ago [2]. Since its discovery in 1993, ADM has been implicated in the pathobiology of several diseases, including cardiovascular disorders, systemic inflammatory responses, sepsis, and septic shock [3]. The interpretation of ADM as a biomarker is difficult because several fragments of the precursor prohormone circulate in blood and do not necessarily reflect the activity of ADM. Several assays for measuring ADM have been described, but they were found to be unreliable, being influenced by the ADM-binding protein (complement factor H) and suffering from the instability of the protein (ADM) [4]. To circumvent previously reported problems with the measurement of ADM, midregional pro-adrenomedullin (MR-proADM) assays have been developed and described as an alternative surrogate marker [5]. Due to the different biological backgrounds, both ADM and mid-regional MR-proADM are derived from the same precursor (pro-ADM), but their stoichiometric relationship is imperfect, as ADM requires a C-terminal amidation to become biologically active adrenomedullin (bio-ADM). Only the amidated-ADM variant, in fact, may bind to its receptor with high affinity and efficiently induce the cAMP formation and related biological activities [6]. However, the C-terminal amidation process occurs only partially and to a varying extent. In addition, the clearance kinetics of bio-ADM and MR-proADM may differ. Taken together, it seems conceptually preferable to measure bio-ADM rather than other derivatives of the ADM precursor peptide and, therefore, in 2017, Weber et al. developed a sandwich immunoassay for bioactive plasma adrenomedullin which has been found to be accurate and reliable and has an analytical sensitivity high enough to be detectable in subjects and patients with various acute inflammatory disorders, including sepsis [7]. The data reported in the paper by Simon et al. show an association between the bio-ADM levels at ICU admission and the severity of acute respiratory distress syndrome (ARDS) in patients suffering from COVID-19, indicating a central role of bio-ADM in the pathology of COVID-19-induced pulmonary injury. In addition, the data highlight the potential of bio-ADM to identify patients in need of invasive ventilation during the ICU stay, as patients with invasive ventilation revealed significantly increased levels of bio-ADM at ICU admission. Furthermore, bio-ADM plasma values were closely related to the need for venovenous extracorporeal membrane oxygenation (ECMO) therapy and renal replacement therapy (RRT) [1]. 

A second article by Ligi et al. deals with the role of circulating histones in COVID-19 [8]. Histones (i.e., positively charged multifunctional nuclear proteins) are key components in chromatin functions, which bind to the nucleosomal core particle around the DNA entry and exit sites. These intriguing molecules may be significantly released in bodily fluids during several targeted organ injuries (e.g., thrombosis, cancer, sepsis, etc.), thus mediating inflammatory pathways and coagulative cascade crucially linked to the severity and mortality of many human pathologies. More recently, they have been reported to be a novel biomarker, which could assist risk stratification in patients with COVID-19 [9] and may serve as a predictive factor for cardiac and lung injury/dysfunction. In addition, they are useful for the individual management of anticoagulant/anti-platelet therapy. The data provided in the already-cited paper, moreover, suggest “the urgent need to clinically evaluate the beneficial role of histone-neutralizing therapy by focused trials involving the interesting roles of polyanionic compounds as potential additional strategy for protecting tissues and organs from inflammatory, cytotoxic and procoagulant effects of circulating histones, implicated in myriad NET- and histone-accelerated disease states, and in COVID-19 complications” [8].

A third paper deals with the role of circulating soluble ST2 (sST2) in COVID-19 [10]. Interleukin-1 receptor-like-1 (IL1RL1), also known as the suppression of tumorigenicity 2 (ST2), is a member of the interleukin (IL)-1 receptor family, with two main isoforms, a transmembrane cellular (ST2L) and a circulating soluble form (sST2). ST2 is the receptor for IL-33, a cytokine released by cells in response to cell damage or stress (‘alarmin’). IL-33 binding to ST2L elicits a pleiotropic action. It is well established that the IL-33/ST2 axis exhibits a cardioprotective role, reducing fibrosis, cardiomyocyte hypertrophy, and apoptosis and improving myocardial function [11]. In their paper, Sánchez-Marteles et al. demonstrated that “admission sST2 values correlate well with biochemical markers and indexes being used to evaluate the clinical course in COVID-19”. Indeed, multivariate regression models and survival analysis demonstrated that admission sST2 is an independent predictor of worse prognosis in patients admitted for COVID-19. In ROC analysis, the sensitivity and specificity of sST2 to predict worse outcomes were significantly higher than that of the aforementioned biomarkers [10]. A unique aspect of the study is “the demonstration that early changes predict outcome better than peak values, stressing their clinical usefulness in prognosis” [10]. Moreover, sST2 showed a decline in patients at discharge which correlates with hospital stay lengths, displaying a fully dynamic behavior that is not present with other biomarkers. 

Another paper published in the journal is about the muscular enzyme creatine (phospho)-kinase (CK/CPK) in patients suffering from COVID-19. The CK levels at admission were higher in those subjects who later experienced more severe outcomes (median, 126; range, 10–1672 U/L, versus median, 82; range, 12–1499 U/L, *p* = 0.01), and values > 200 U/L (hyperCKemia) were associated with a worse prognosis. Regression analysis confirmed that increased CK acted as an independent predictor for a “severe” outcome. HyperCKemia was generally transient, returning to normal during hospitalization in the majority of both “severe” and “mild” patients [12]. The study design did not allow the authors to understand if increased CK levels in COVID-19 patients are caused by true myopathic damage, as muscle pain and fatigue are common in both mild and severe cases [12]. Further studies are, therefore, needed to better understand the pathophysiology of increased release of CK and other muscular enzymes (e.g., aldolase). 

On the one hand, these papers highlight the formidable advancements in our knowledge of the pathophysiological mechanisms of COVID-19 and the role of innovative biomarkers in risk stratification and disease prognostication. One the other hand, they reinforce the need for further translational research studies to appropriately use these biomarkers in clinical practice to ultimately improve patient care.
